# Learning from Persistent Viremia: Mechanisms and Implications for Clinical Care and HIV-1 Cure

**DOI:** 10.1007/s11904-023-00674-w

**Published:** 2023-11-13

**Authors:** Fengting Wu, Francesco R. Simonetti

**Affiliations:** Division of Infectious Diseases, Department of Medicine, Johns Hopkins University School of Medicine, 733 N Broadway, Baltimore, MD 21205 USA

**Keywords:** Residual viremia, Nonsuppressible viremia, Clonal expansion, Defective provirus, HIV-1 integration, Virus production

## Abstract

**Purpose of Review:**

In this review, we discuss what persistent viremia has taught us about the biology of the HIV-1 reservoir during antiretroviral therapy (ART). We will also discuss the implications of this phenomenon for HIV-1 cure research and its clinical management.

**Recent Findings:**

While residual viremia (RV, 1–3 HIV-1 RNA copies/ml) can be detected in most of people on ART, some individuals experience non-suppressible viremia (NSV, > 20–50 copies/mL) despite optimal adherence. When issues of drug resistance and pharmacokinetics are ruled out, this persistent virus in plasma is the reflection of virus production from clonally expanded CD4^+^ T cells carrying proviruses. Recent work has shown that a fraction of the proviruses source of NSV are not infectious, due to defects in the 5′-Leader sequence. However, additional viruses and host determinants of NSV are not fully understood.

**Summary:**

The study of NSV is of prime importance because it represents a challenge for the clinical care of people on ART, and it sheds light on virus-host interactions that could advance HIV-1 remission research.

## Introduction

**Fig. 1:**
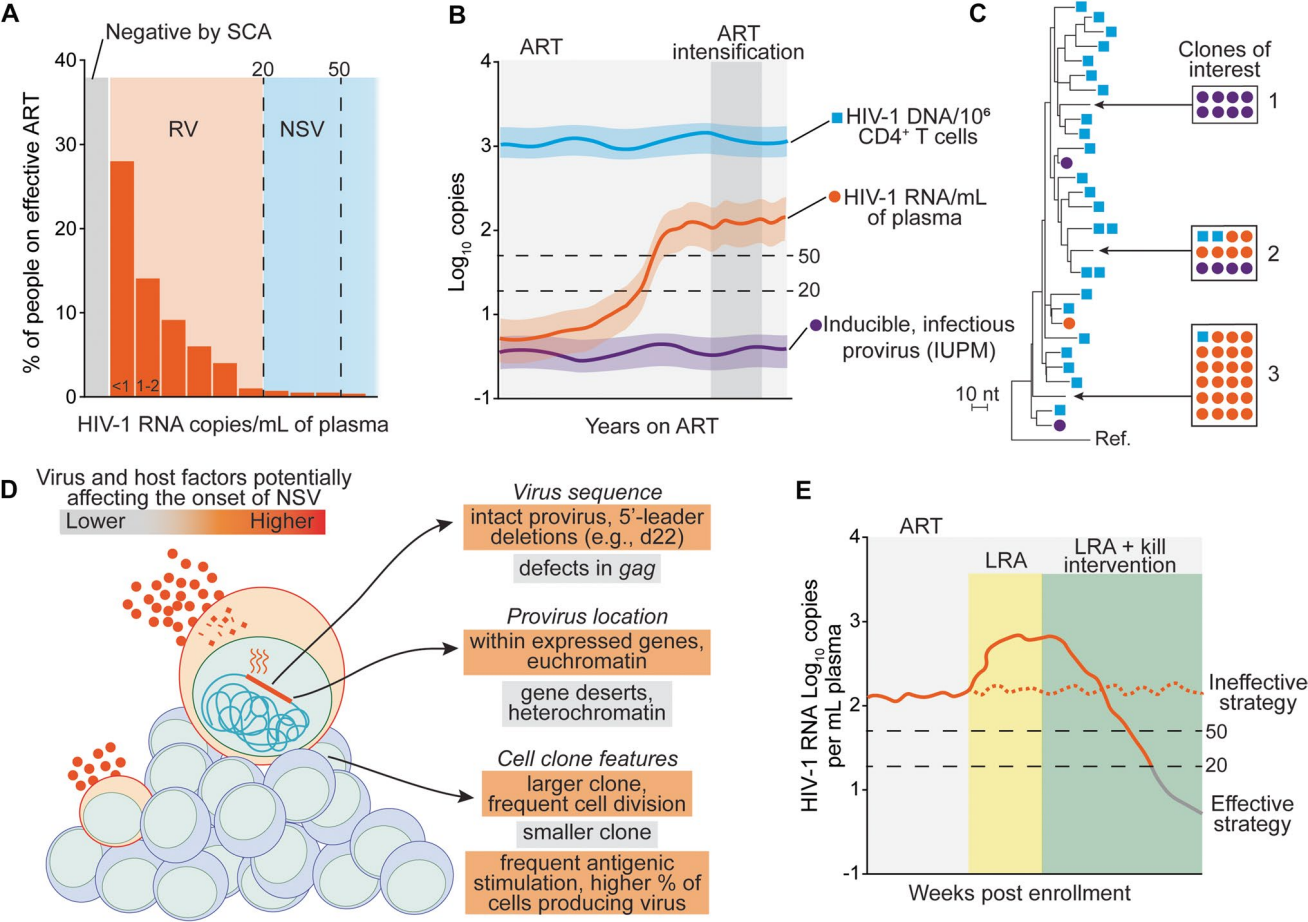
Characteristics of persistent HIV-1 viremia during effective antiretroviral therapy. **A** Distribution of HIV-1 RNA levels in plasma among people on ART; each bar represents a range of HIV-1 RNA copies/mL; RV, residual viremia; NSV, non-suppressible viremia; SCA, single copy assay; limit of detections of most clinical assays are indicated by dashed lines. **B** Example of an individual developing NSV after years of undetectable viremia; standard ART regimen and ART intensification and indicated by light and dark gray areas, respectively. **C** Representative simulated phylogenetic maximum-likelihood tree of HIV-1 sequences recovered from plasma virus (orange circles), viral outgrowth (purple circles), or proviral DNA (blue squares); boxes and arrows indicate large groups of identical sequences reflecting clonally expanded HIV-1-infected cells; example clones of interested are indicated by numbers: clone 1 is replication competent but does not contribute to viremia; clone 2 is also infectious, it can be found in plasma, and it can be detected among infected cells; clone 3 is a predominant plasma variant, but it cannot be recovered by viral outgrowth. **D** Summary of virus and host factors that may favor only some infected clones to contribute to NSV. **E** Schematic of the potential response of a study participant with NSV to experimental interventions aimed at enhancing virus production and/or elimination of infected cells causing NSV; LRA, latency-reversing agent

During acute infection with human immunodeficiency virus type 1 (HIV-1), plasma HIV-1 RNA (commonly referred to as viral load, VL) reaches a peak of ~ 10^6^ copies/mL before falling to a lower set point of ~ 10^4^ copies/mL [1]. Untreated HIV-1 infection progresses to acquired immunodeficiency syndrome (AIDS) as peripheral blood CD4^+^ T cells decrease to below 200 cells/uL [2], eventually leading to life-threatening opportunistic infections and cancer. Antiretroviral therapy (ART) halts viral replication by inhibiting multiple steps of the HIV-1 life cycle. Blockade of viral replication rapidly causes a biphasic, exponential decrease of plasma VL to below the limit of detection of clinical assays (20–50 copies/mL) [3, 4]. Maintaining undetectable VL results in CD4^+^ T cell recovery, arrest of disease progression, and prevents HIV-1 transmission [5, 6]. Despite its success in changing the history of the epidemic, ART can only block infection of new target cells and does not affect latently infected cells containing stably integrated HIV-1 DNA (provirus) [7, 8]. A small fraction of these cells called the HIV-1 reservoir carries infectious proviruses that can rekindle viral replication shortly after ART interruption [9–11]. Another reflection of HIV-1 persistence despite years of ART is the presence of low levels of HIV-1 RNA in plasma due to the daily activation of latently infected cells leading to virus production (Table 1). Indeed, ultrasensitive assays and intensive sampling of plasma revealed that about half of people living with HIV (PLWH) on ART have traces of HIV-1 RNA, referred to as residual viremia (RV), of about 1–3 copies/mL (Fig. 1A) [12••, 13••]. Some PLWH may experience viral blips, isolated episodes of detectable VL followed by re-suppression [14, 15]. Moreover, some individuals present with or develop persistently or intermittently detectable low-level viremia (LLV), usually below 200 copies/mL, that is not due to drug resistance or problems with adherence or drug absorption. In recent years, the term non-suppressible viremia (NSV) has been used *in lieu* of LLV [16••]. The term NSV shifts the focus on the fact that this viremia, regardless of the amount of virus measured by clinical assays, is not due to viral replication and cannot be further decreased by modification or intensification of ART. For example, we recently described an individual with NSV caused by a single non-infectious provirus, persistently around 3 × 10^3^ copies/mL [17••]. To a certain extent, RV and NSV are reflections of the same phenomenon: spontaneous transcriptional activity of HIV-1 infected cells (Fig. 1A and B). In this review, we present the recent developments regarding the characteristics and drivers of RV and NSV in people on effective ART and discuss unmet knowledge gaps that would provide a better understanding of HIV-1 host interactions and clinical management.

**Table 1:** Definitions of HIV-1 RNA in plasma during ART

Definitions of HIV-1 RNA in plasma during ART	Detected by clinical assays	Detected by ultrasensitive assays	HIV-1 RNA copies per mL of plasma	Notes
Residual viremia (RV)	no	yes	~ 1–3	Reflection of HIV-1 persistence; caused by release of viral particles from infected cells
Low level viremia (LLV)	yes	yes	variable*	
Non-suppressible viremia (NSV)	yes	yes	> 20	NSV not caused by virus replication, It is an extreme form of RV
Viral blips	yes, isolated measurements	yes	< 1000	
Virological failure (VF)	yes	yes	variable**	Result of overt viral replication due to drug resistance or insufficient drug concentrations

^*^JAMA/International AIDS Society: 50-200cp/mL, WHO: 50-1000cp/mL

^**^JAMA/International AIDS Society: 2 consecutive VL > 200cp/mL, WHO: 2 consecutive VL > 1000cp/mL

## Lessons Learned from Residual Viremia

The presence of RV during effective ART has been recognized more than two decades ago owing to the development of sensitive PCR assays based on ultracentrifugation of plasma [12••, 13••]. Ultracentrifugation not only concentrates viral particles, increasing sensitivity, but also narrows downstream analyses to packaged, membrane-associated HIV-1 RNA. These single-copy assays evolved over time, with optimizations that allowed greater recovery and reduced amplification failure due to inter-individual viral diversity [18]. A newer version of this protocol pellets virus with a table-top centrifuge, avoiding the need for ultracentrifugation [19]. Finally, automated extraction and amplification systems based on multi-replicate analyses perform equally to manual approaches and allow the study of larger sample sets and participant cohorts [20].

The quantitative and qualitative characterization of RV has been instrumental in the understanding of HIV-1 persistence. However, the root mechanisms of RV have been one of the most debated topics in HIV-1 biology: is RV caused by ongoing viral replication, or is it the result of virus production from a pool of persistent cells infected before ART? Understanding which of these two mechanisms contributes to RV and reservoir maintenance has profound implications, as it dictates whether the development of curative strategies should focus on antiretrovirals with better potency and tissue penetration, or the elimination of latently infected cells [21]. Despite some reports describing cryptic HIV-1 replication in anatomical sites (central nervous system, genital tract, and gut) due to low drug penetration, there is vastly preponderant evidence supporting that RV is caused by the release of virus from expanded cells rather than ongoing viral replication [22–27]. Since HIV-1 reverse transcriptase is highly error-prone and has a striking propensity for recombination [28], ongoing viral replication should result in a diverse population of viruses in plasma that accumulates new mutations over time [29]. However, longitudinal sampling indicated viral populations lacking sequence diversity and evolution, suggesting that RV must be the result of virus production from cells infected before ART introduction, rather than from new infection events [30]. In most individuals, one or few predominant plasma sequences are identified, reflecting one or few infected T cell clones contributing to residual viremia [31••]. These so-called predominant plasma clones (PPC) are non-evolving and sensitive to the concurrent drug regimen, further supporting that RV is not due to ongoing viral replication. In addition, attempts at ART optimization, or intensification by adding a fourth drug, are not effective [32, 33]. HIV-1 RNA sequences from plasma often, but not always, match rare proviral genomes from peripheral blood CD4^+^ T cells and tissues [31••, 34]; this observation represented the first evidence that infected cells persist during long-term ART through cell proliferation, which is now recognized as the major mechanism of reservoir persistence and a barrier to HIV-1 remission [31••, 35, 36, 37••, 38, 39]. Similarly to infected T cell clones, the frequency and relative abundance of plasma viruses also wax and wane over time on ART, suggesting a correlation between the stimuli driving clonal dynamics and virus production from infected cells [31••, 34, 40]. However, the exact mechanisms by which the cells responsible for RV are induced to produce virus remain unclear.

To better understand the source and consequences of HIV-1 persistence, numerous studies investigated clinical and laboratory parameters that could correlate with RV. Persistent viremia on ART positively correlates with HIV-1 RNA levels before starting ART, initiation of treatment during the chronic phase of infection, and, in some studies, with the frequency of infected cells based on total proviral DNA [14, 41]. In an analysis of paired samples from individuals who had been on ART for 24–96 weeks, RV had a moderate yet significant correlation with reservoir size by the quantitative viral outgrowth assay (qVOA) [42]. These results suggested that, to some extent, RV reflects the magnitude of proviruses that persist upon ART introduction. However, a striking majority of proviruses have defects that preclude the formation of viral particles and/or replication competence, complicating the association between RV and cell-associated HIV-1 DNA and RNA [43]. Additional studies showed that RV is higher—or more likely to be detected—in individuals who have been on ART for less than 2 years, are male, have higher body mass index, and are older [44, 45]. In a large longitudinal study, Riddler and colleagues investigated the dynamics of persistent RV from participants who had been on suppressive ART between 4 and 12 years. This work revealed a continued decline of RV over time (6% per year), with an estimated half-life of 11 years, suggesting a progressive loss of infected cells capable of producing virus [46]. Such observation was consistent with the known slow decay of the reservoir [47••] and foreshadowed more recent studies showing that intact proviruses decay faster than those with defects [48–53] and that the reservoir is progressively enriched in genomic locations associated with deeper viral latency [54].

## Detectable Viremia Despite Optimal Adherence

NSV represents an extreme case of RV, in which HIV-1 RNA is persistently—or intermittently—above the limit of detection of clinical assays (> 20–50 copies/mL) despite 100% adherence to ART (Fig. 1B). In one of the first reports, Simonetti et al. described an individual who developed NSV after the diagnosis of oral cancer [55]. Plasma virus had two sources: (i) a swarm of replicating, drug-resistant variants that disappeared after ART optimization and (ii) a single, drug-sensitive PPC. The latter variant decreased immediately after chemo-radiation but reappeared upon cancer recrudescence and disease progression. Viral outgrowth experiments revealed that the virus in plasma was replication-competent, harbored by a single CD4^+^ clone that underwent extensive proliferation. These results, which contradicted previous data suggesting clonally expanded T cells contain only defective proviruses [56], were then confirmed by multiple studies [35, 36, 38]. Analysis of CD4^+^ T cells from multiple anatomical sites demonstrated enrichment in metastatic tissues of the provirus causing NSV called “AMBI-1” because of its ambiguous genomic location. These results led to the hypothesis that infected CD4^+^ T cells from this clone were producing viruses in response to a cancer neoantigen. The characteristics of NSV were then confirmed by the description of the same phenomenon in a small cohort of people on ART by Halvas and colleagues [16••]. In most individuals, NSV appears in the absence of clinical events or changes in therapy, usually after years of undetectable VL [16••, 17••]. Although only a few studies with small sample sizes are available, we roughly estimate that about 1 in 250 individuals on effective ART experiences NSV [16••, 17••]; larger, multi-center studies are needed to provide a better frequency of this phenomenon. As with RV, the characterization of plasma virus from individuals with NSV shows no drug resistance mutations to concurrent ART and drug concentrations within the therapeutic range [16••, 17••]. Similarly, NSV is sustained by one or a few PPCs (Fig. 1C) [16••, 17••]. The development of NSV is likely multifactorial, a “perfect storm” of both viral and host processes. Interestingly, if these factors are present in all people on ART, why do only rare individuals experience persistent NSV? We will further address some of these mechanisms in the section below, summarized in Fig. 1D.

### Proviral Sequence

A fraction of HIV-1 variants in plasma can be recovered from viral outgrowth ex vivo (for example, clone 2 in Fig. 1C), indicating replication-competence of the proviruses giving rise to NSV [16••, 57, 58]. In addition, previous studies linked clones contributing plasma viremia and cell-associated RNA to viral rebound sequences in the context of analytical treatment interruption (ATI) [58–60]. However, not all PPCs can be isolated from viral outgrowth assays (e.g., clone 3 in Fig. 1C), which could be explained by limited sampling, tissue residency of infected clones, or the inability of these variants to infect target cells. Defective HIV-1 genomes represent approximately 95% of the proviruses found in people on ART [48, 61••]. Some defective proviruses can produce HIV-1 RNA and proteins, resulting in the expression of viral antigens and viral particles [62, 63]. The in-depth characterization of 4 individuals with NSV revealed defective viruses with small 5′-Leader defects, typically 21–22 nucleotide deletion or mutations affecting the Major Splicing Donor (MSD) site [17••]. The secondary RNA structure of 5′-Leader orchestrates multiple steps in the HIV-1 life cycle: initiation of reverse transcription, transactivation of proviral transcription, dimerization of genomic RNA, binding to Gag, packaging in viral particles, and alternative splicing of viral mRNA [64–67]. Thus, even small defects in the 5′-Leader can abrogate viral replicative fitness [48, 61••]. Interestingly, three out of four participants shared the same 22-nucleotide deletion (d22). In a separate study, three out of eight cases of NSV were also caused by proviruses with 5′-Leader defects, one affected by the same d22 deletion [68]. These recurring deletions may be favored by repeated sequences that cause a misplaced template switch by the reverse transcriptase. Although proviruses with 5′-Leader deletions are common, representing about 5–10% of all proviruses found during suppressive ART [17••, 48, 57, 61••], the d22 deletion is extremely rare (Duan et al., in preparation). Since this specific defect can be found in the plasma of multiple participants, we posit that some defects, like d22, can promote the development of NSV. We recently demonstrated that deletions in the MSD can result in new strong splice donors that only partially rescue alternative splicing, resulting in the production of viral particles lacking Envelope incorporation [17••, 69]. Future studies should investigate whether these 5′-Leader defects provide a selective advantage. The lower Envelope expression and viral particle production could result in decreased cytopathic effect and escape from neutralizing or effector antibodies, extending the half-life of virus-producing cells.

### Proviral Location

The site of HIV-1 integration can influence the persistence of infected cells and viral gene expression. Proviruses located within gene bodies can affect the expression of the surrounding gene, a phenomenon known as insertional mutagenesis [70]. For example, proviral promoters can increase host gene mRNA expression (as in the case of *BACH2* and *STAT3*), while the introduction of splice sites and polyadenylation signals can disrupt host genes [70, 71••]. While these processes can lead to loss of infected cells if detrimental to T cell function, HIV-1 integration in rare genes (*BACH2*, *STAT5B*, *MKL2*, *MKL1*, *IL2RB*, *MYB*, and *POU2F1*) has been linked to the persistence of a small fraction of infected cells in people on ART [72••]. Growing evidence suggests that the genomic context of a provirus may affect its expression and the survival of the infected cells over time. A few studies reported an accumulation of intact proviruses in heterochromatic chromosomal locations, such as centromeric alpha repeats, and zinc finger (ZNF) gene clusters in elite controllers, people who have been on ART for multiple decades, and post-treatment controllers [54, 73–75]. Since HIV-1 integration favors easily accessible actively transcribed genes, proviruses in transcriptionally inactive regions of the genome are rare before or shortly after ART introduction [76, 77]. Thus, the observed enrichment is likely the reflection of a selective advantage: the protection from adaptive and innate immunity via deeper latency. Due to the limited sample size, whether genomic location plays a key role in proviruses that cause NSV is still unclear. However, the majority of proviruses reported so far were found within introns of genes with variable expression in CD4^+^ T cells and all integrated into the opposite orientation of the gene transcription (*MATR3* [16••], *ADK*, *DNAJB14*, *RRM1*, *ZFYVE9*, *CCND3* [17••]). Recently, Mohammadi and colleagues reported that virus-producing proviruses were enriched in proximity to the activating H3K36me3 epigenetic mark [68]. Taken together, the current evidence suggests that an epigenetic environment permissive to latency reversal and HIV-1 transcription can contribute to the development of NSV.

### Clonal Expansion

Infected CD4^+^ T cells proliferate through a combination of mechanisms, including the effects of HIV-1 integration (as discussed above), homeostatic stimuli, and antigen-driven proliferation [78]. To recall immune responses against previously encountered pathogens, memory cells need to be maintained even in the absence of antigenic exposure [79]. Homeostatic proliferation and survival of T cells are driven by cytokines such as IL-7 and IL-15 [80–84]. Indeed, these cytokines also play a role in the persistence of infected CD4^+^ T cells. Higher levels of IL-7 in plasma are correlated with a higher frequency of proliferating cells in PLWH [85]. Of note, IL-7 allows cell division with little to no production of viral particles, allowing the proliferation of infected cells without recognition by immune surveillance [86, 87]. Several recent studies have shown that antigen-driven proliferation in PLWH contributes to the expansion and selection of infected cells [37••, 57]. Although the size of the latent reservoir is stable, individual clones of infected cells comprising the reservoir are far more dynamic, as they tend to wax and wane over months or years [40]. The process of waxing and waning is the reflection of clonal expansion and contraction, typical of immune responses to antigens. Infected cells contributing to NSV are usually detected as part of expanded clones, but the cell dynamics that preceded the onset of NSV are poorly understood. In one participant described by White, Wu et al., the provirus responsible for 100% of plasma virus was undetectable before viremia became clinically relevant and subsequently reached a new plateau of ~ 50 copies/10^6^ CD4^+^ T cells. Whether this increase was the result of gradual inflation over time or a rapid burst of divisions in response to antigenic stimulation remains unclear. Although specific sequences dominate NSV, the proviruses from which they are derived are rare among all proviral variants (e.g., clone 3 in Fig. 1C). Harnessing integration site-specific digital PCR, White, Wu et al. showed that proviruses source of NSV were compartmentalized in effector memory cells [17••]. Given the shorter half-life of this more differentiated subset, these clones must be maintained by frequent proliferation [88]. Finally, although the overall size of an infected clone likely contributes to the development of NSV, clonal size does not correlate with the magnitude of viremia, suggesting that other processes are at play. Some factors could include which fraction of a clone produces virions at a given time, the amount of virus produced per cell before it dies or returns to latency, and the frequency with which the cells of a clone are stimulated.

### Immune Stimulation

The exact mechanisms contributing to the activation of clonally expanded cells causing NSV are poorly understood. As depicted in Fig. 1C, only one or a few variants contribute to persistent NSV, while viruses derived from other inducible, infectious proviruses, abundant among those recovered by viral outgrowth, are not found in plasma (clone 1). The activation of an infected CD4^+^ T cell is necessary for the completion of HIV-1 RNA expression and virion production [89]. We posit that chronic or ubiquitous antigens could be the immune stimuli inducing spontaneous reservoir activity, leading to RV and NSV [90]. Antigens are processed and presented as peptides on major histocompatibility complex (MHC) molecules on the surface of antigen-presenting cells and recognized by the T cell receptor (TCR) on T cells [91]. The TCR-peptide-MHC interaction triggers downstream pathways transcriptional activation, effector function, and expansion of T cells [92]. In addition, self-peptides loaded on MHC molecules provide survival stimuli and tonic signaling to maintain proper reactivity to foreign antigens [93, 94]. Our group recently reported that proviruses causing NSV can be found in cells responsive to viral and autologous protein stimulation [95]. The frequency of CD4^+^ T cells that recognize such antigens and also carry a provirus capable of producing viral particles is extremely low, potentially explaining the low frequency of NSV among people on ART.

## Consequences of Persistent Viremia

People on long-term ART, even with undetectable viremia and optimal CD4^+^ T cell recovery, show higher levels of inflammatory markers than HIV-1 negative controls [96]. The drivers of this phenomenon are complex and include co-morbidities, other chronic infections (one above all, cytomegalovirus), lifestyle, residual viral expression from HIV-1-infected cells, and legacy effects from the period of untreated infection [97, 98]. However, to which extent RV and NSV drive immune activation and chronic inflammation remains unclear. The presence of RV is associated with older age, potentially reflecting the weakening of immune responses, the inflation of the memory T cell compartment, and the progressive clonal expansion of total and HIV-infected cells[99–103]. Some groups reported the association of RV with increased levels of soluble IL-6, CD14, and TNF-ɑ as an indication of chronic immune activation [104–106], while others found no association with IL-6, but rather microbial translocation [107, 108]. Riddler et al. reported that RV is associated with higher CD8^+^ T cell counts and a lower CD4/CD8 ratio on ART, both markers suggesting incomplete immune recovery [46]. However, a direct link between RV and NSV remains elusive, because it is challenging to untangle whether inflammation is caused by pre-ART immune dysregulation or directly due to persistent viremia on ART. In a study by Gandhi et al., the higher levels of immune activation in people on suppressive ART were associated with higher levels of inflammation and set-point viremia before ART introduction, suggesting that immune dysregulation is a long-term sequelae, rather than an ongoing process sustained by persistent viremia [109••]. One way to tease out the contribution of RV and NSV would be to monitor immunological markers before and after an experimental intervention that effectively reduces HIV-1 expression and viral particle formation. Unfortunately, as discussed below, such intervention is not currently available, and research efforts in this direction remain a high priority.

## Implications for Cure Strategies

The ultimate goal of achieving an HIV-1 cure is to significantly reduce the reservoir size and induce immune control that would prevent viral rebound, disease progression, and transmission in the absence of ART [110, 111]. The mechanisms driving RV and NSV reflect the major challenges in finding a cure for HIV-1 for all people on ART. If the proviruses contributing to viral rebound are those with frequent transcriptional activity, then proviruses responsible for RV and NSV may be the first to rekindle HIV-1 replication upon ART cessation. Indeed, proviral sequences found in some people on ART are identical to plasma viral sequences found before and after ATI, suggesting that a fraction of variants from RV can also lead to viral rebound [58, 59, 112]. However, without knowing the immune stimuli driving the activation of these infected clones, the exact link between viruses contributing to RV and those causing rebound remains unclear. Rebound-competent proviruses may have acquired mutations that allow for escape from immune pressure, such as autologous neutralizing and effector antibodies, cytolytic T cells, and interferon [113, 114]; thus, understanding the replicative fitness and immune escape profile of variants causing persistent viremia should be thoroughly investigated in future studies.

One of the main strategies pursued to eliminate the HIV-1 reservoir is the so-called “shock-and-kill,” in which latency reversal is followed by enhanced immune engagement and killing of virus-producing cells [110, 111]. Unfortunately, despite promising pre-clinical data, most latency-reversing agents (LRAs) tested so far are ineffective at inducing robust levels of viral antigen expression from infected cells in vivo, preventing “killing” strategies from reaching their full potential [115, 116]. The presence of NSV is often an exclusion criterion for enrollment into HIV-1 cure clinical trials. The main rationale is that individuals with persistent viremia may not be fully suppressed or have an atypical reservoir size and composition. In addition, the presence of detectable viremia could further complicate the primary outcome analyses, such as viral load changes upon ATI. We argue that clinical trials involving participants with NSV could offer the opportunity to study novel interventions to reactivate and eliminate reservoir cells or even proviral latency (the so-called “block and lock”) [117]. The lack of an effective LRA to induce HIV-1 expression without global T cell activation is a great roadblock to test effective kill strategies in clinical trials; thus, the selective elimination of infected clones with spontaneous virus production, like those fueling NSV, could be used to test the efficacy of an intervention in vivo. In addition, given the short half-lives of both virions and virus-producing cells, the impact of shock-and-kill strategies could be assessed rapidly by monitoring changes in viral load, without the need for ultrasensitive single-copy assays or ATIs to understand whether an intervention effectively killed the HIV-1-infected clones of interest (Fig. 1E).

Recent discoveries showed that the inflammasome molecule CARD8 can sense HIV-1 protease activity in virus-producing T cells and macrophages [118]. HIV-1 eludes this innate mechanism because protease is mostly active only after viral particles leave the cell [119]. However, some non-nucleoside reverse transcriptase inhibitors (NNRTIs) that have been used for two decades to treat HIV-1, such as efavirenz and rilpivirine, have recently been shown to induce intracellular Gag-Pol dimerization and premature protease activation, triggering the CARD8 inflammasome, and death of HIV-1-producing cells via pyroptosis [118, 120]. In the context of RV and NSV, the use of NNRTIs or other molecules inducing CARD8-sensing of HIV-1 could represent a promising strategy to eliminate infected cells contributing to persistent viremia and accelerate reservoir decay [121].

## Clinical Management of NSV

The onset of NSV after years of undetectable VL is a challenging clinical scenario. In the past, larger cohort studies have linked detectable viremia with a higher risk of virological failure and selection for drug resistance [122••, 123–125]. However, in recent studies with in-depth virological characterization, individuals did not experience viral rebound or selection for drug-resistance mutations when viremia is caused by virus production from infected clones [16••, 126]. NSV can also be a cause of concern regarding transmission, especially when HIV-1 RNA levels are above 200 copies/mL, since landmark studies demonstrated that undetectable equals untransmissible (U = U) for people on ART with < 200 copies/mL [127••]. However, a recent systematic review showed almost zero risk for sexual transmission when VL is less than 1000 copies/mL, supporting that U = U is also for people with NSV [128].

Determining whether detectable viremia is due to an incomplete blockade of viral replication or due to virus release remains critical for the correct management of people on ART with detectable viremia. Due to the lack of unified guidelines and technical limitations, understanding the cause of NSV is challenging [122••]. Table 2 summarizes the causes, consequences, and management of detectable viremia. Importantly, the lack of awareness surrounding NSV often leads to unnecessary tests, treatment changes, and mistrust when persons report optimal adherence, compromising the relationship between clinical care providers and patients. If the cause of persistent viremia is not understood, NSV can be a source of frustration and anxiety on both sides.

**Table 2:** Clinical management of detectable viremia on ART

Machanism underlying detectable viremia on ART	Causes	Consequences	Management
*Viral replication* (ongoing new infection events)	Suboptimal adherence	Viral evolution	Adherence counselling
	Reduced ART concentration due to drug-to-drug interactions or limited absorbsion	Drug resistance	Therapeutic drug monitoring
		Increase in viremia and virological failure	HIV-1 genotyping from plasma***
		CD4^+^ T cells loss	ART regimen optimization
	Drug resistance	Risk of transmission	
*Viral expression* from infected cells (virus production)	Latency reversal resulting in continuous virus production	No viral evolution	If all of the above not suggestive for viral replication:
		No drug resistance to current regimen	Monitoring of viremia over time
		Intermittent or stable viremia for months/years	Short course ART intensification
	Clonal expansion of cells carrying intact or defective proviruses	Stable CD4^+^ T cells	***Characterization of virus in plasma with ultrasensitive assays***
		Potential higher inflammation	

^***^Usually possible only with HIV-1 RNA levels above 400 copies/mL

Sequencing of plasma virus remains the most informative step to guide the management of detectable viremia. Unfortunately, clinical-grade genotyping assays often fail when plasma HIV-1 RNA is below 500 copies/mL. Consequently, clinicians usually resort to genotyping HIV-1 DNA from infected cells; although this approach is successful even when viremia is undetectable, bulk sequencing of HIV-1 DNA hardly represents the rare proviruses causing NSV [129, 130]. Thus, there is a dire need to develop ultrasensitive clinical assays that can sequence low-level plasma virus and provide information regarding (*i*) the presence of drug resistance, (*ii*) defects that would abrogate infectiousness, (*iii*) degree of clonality, and (*iv*) the evolution—or lack thereof—of plasma variants over time.

Currently, there is no effective pharmacological strategy to further decrease viremia in people on effective ART, as antiretrovirals only block new infection events of target cells. For example, fostemsavir, an attachment inhibitor that prevents the binding of gp120 to CD4 molecules, is a newly FDA-approved drug with shown efficacy in PLWH with multidrug resistance [131, 132]. However, since fostemsavir has no effect on virus production, it cannot reduce HIV-1 RNA expressed from persistent reservoirs. In a recent study, fostemsavir intensification in a participant with NSV did not decrease plasma viral load [17••]. Lenacapavir is a new-in-class long-acting capsid inhibitor that blocks core disassembly, the interaction between capsid and host factors, viral assembly, and maturation [133]. Lenacapavir has been recently approved in Europe and North America for the treatment of multidrug-resistant HIV-1 infection [134]. At higher concentrations, lenacapavir inhibits capsid assembly and viral particle formation in vitro, which may reduce HIV-1 RNA in plasma. Future studies should investigate whether ART intensification with lenacapavir could be a successful strategy to treat individuals with NSV.

## Concluding Remarks

Since the discovery of the latent reservoir, the characterization of viremia has played a key role in the understanding of HIV-1 persistence, from viral dynamics upon ART introduction to the proliferation of infected clones [13••, 31••]. Decades later, we are still learning from RV and NSV, and remarkable progress has been made in defining which virus and host factors shape the selection of long-lived infected cells. However, numerous questions remain unanswered, including which processes lead to spontaneous proviral expression of only selected cells, which fraction of viruses in plasma can cause viral rebound if ART is stopped, and whether persistent viremia sustains ongoing immune stimulation. Moreover, there is a critical need for better clinical assays, guidelines, and therapeutic options to assist those individuals with detectable viremia despite 100% adherence to ART. The study of HIV-1 viremia remains an ideal nexus between basic and clinical science. Further research surrounding RV and NSV is needed, as not only it can provide new insight into reservoir persistence and how to perturb it but it can also improve the clinical care of people living with HIV-1.

## Data Availability

Not applicable.
